# Unexpected Course of Nonlinear Cardiac Interbeat Interval Dynamics during Childhood and Adolescence

**DOI:** 10.1371/journal.pone.0019400

**Published:** 2011-05-20

**Authors:** Dirk Cysarz, Maijana Linhard, Friedrich Edelhäuser, Alfred Längler, Peter Van Leeuwen, Günter Henze, Georg Seifert

**Affiliations:** 1 Integrated Curriculum for Anthroposophic Medicine, University of Witten/Herdecke, Witten, Germany; 2 Chair for Theory of Medicine, Integrative and Anthroposophic Medicine, University of Witten/Herdecke, Witten, Germany; 3 Department of Pediatric Oncology/Hematology, Otto-Heubner-Center for Pediatric and Adolescent Medicine, Charité Universitätsmedizin, Berlin, Germany; 4 Department of Pediatric and Adolescent Medicine, Gemeinschaftskrankenhaus, Herdecke, Germany; 5 Department of Radiology and Microtherapy, University of Witten/Herdecke, Bochum, Germany; Queensland Institute of Medical Research, Australia

## Abstract

The fluctuations of the cardiac interbeat series contain rich information because they reflect variations of other functions on different time scales (e.g., respiration or blood pressure control). Nonlinear measures such as complexity and fractal scaling properties derived from 24 h heart rate dynamics of healthy subjects vary from childhood to old age. In this study, the age-related variations during childhood and adolescence were addressed. In particular, the cardiac interbeat interval series was quantified with respect to complexity and fractal scaling properties. The R-R interval series of 409 healthy children and adolescents (age range: 1 to 22 years, 220 females) was analyzed with respect to complexity (Approximate Entropy, ApEn) and fractal scaling properties on three time scales: long-term (slope β of the power spectrum, log power vs. log frequency, in the frequency range 10^−4^ to 10^−2^ Hz) intermediate-term (DFA, detrended fluctuation analysis, α_2_) and short-term (DFA α_1_). Unexpectedly, during age 7 to 13 years β and ApEn were higher compared to the age <7 years and age >13 years (β: −1.06 vs. −1.21; ApEn: 0.88 vs. 0.74). Hence, the heart rate dynamics were closer to a 1/f power law and most complex between 7 and 13 years. However, DFA α_1_ and α_2_ increased with progressing age similar to measures reflecting linear properties. In conclusion, the course of long-term fractal scaling properties and complexity of heart rate dynamics during childhood and adolescence indicates that these measures reflect complex changes possibly linked to hormonal changes during pre-puberty and puberty.

## Introduction

The fluctuations of the cardiac interbeat series exhibit a rich complexity because this series is modulated by different physiological functions under neural control such as respiration, blood pressure control or diurnal variations of activity [1]. Taking into account that each modulating function has specific temporal fluctuations (e.g. specific mean frequency and correlation properties) this series reflects the integration of many different characteristics of fluctuations and thus shows chaotic and multifractal properties [2], [3]. Consequently, approaches based on linear as well as nonlinear dynamics must be used to uncover different aspects of this integration. For example, spectral analysis of heart rate variability (HRV) quantifies sinus wave-like oscillations of the cardiac interbeat series [4] whereas Approximate Entropy (ApEn) and Sample Entropy quantify irregularity (complexity) [5], [6], and detrended fluctuation analysis (DFA) quantifies fractal properties (self-similarity on different time scales) analogous to spatial self-similarity [7], [8]. The homeostatic model is insufficient to explain the different aspects. Hence, a homeodynamic view of physiological functions is required [9].

In healthy subjects the dynamics of the cardiac interbeat interval series varies from childhood to old age [10]–[13]. Linear properties of the cardiac interbeat series, i.e. the R-R interval series, such as the standard deviation of normal-to-normal intervals (SDNN) in the time domain or low frequency oscillations in the frequency domain, increase during childhood and adolescence and show a maximum at the age of approximately 30 years [10]. These measures are associated with the increase of the mean R-R interval during childhood [14]–[16]. It has been supposed that the variation during childhood and adolescence is related to the maturation of the different regulatory mechanisms and determinants (e.g. autonomic nervous system, ANS) whereas the variation at older ages reflects their deterioration [10].

Nonlinear measures corresponding to e.g. fractal properties or irregularity seem to show a different variation with age compared to measures reflecting linear properties. Previous work suggests that complexity and fractal properties are constant during childhood and adolescence [10]. Only after about 30 years of age do clear variations over time become apparent. These results suggest that measures derived from nonlinear dynamics are not affected during childhood in spite of the hormonal changes associated with the timing of pre-puberty and puberty [17], [18].

The variations of the R-R interval series during childhood and adolescence with respect to complexity and self-similarity have not as yet been studied thoroughly. Previous results suggest that linear measures of R-R interval dynamics increase during childhood and adolescence whereas nonlinear measures seem to be constant or even decrease [10]. However, the number of subjects in this age range was too low to yield definite conclusions. Other studies only partially covered the age range of childhood and adolescence [15] or did not investigate nonlinear measures [16]. It is not clear whether the dramatic maturation that occurs during childhood and adolescence (accompanied by a vulnerability with respect to the onset of different diseases [19]–[23]) is also reflected by specific linear or nonlinear aspects of cardiovascular regulation. Therefore, the aim of this study was to investigate the maturation of the ANS by means of variations of R-R interval dynamics derived from 24-hour ECG recordings during childhood and adolescence. We will show that in particular nonlinear measures reflecting irregularity and fractal properties show an unexpected development with age.

## Methods

### Ethics Statement

Written informed consent was obtained from the child's guardian and, if applicable, also from the child in accordance with the Declaration of Helsinki. The study protocol was approved by the Ethics Committee of Charité - Universitätsmedizin Berlin.

### Subjects

A total of 469 children and adolescents were initially enrolled in this cross-sectional study. 409 subjects (age range 1 to 22 years, 220 females, 189 males) had an ECG recording suitable for further analysis comprising wake and sleep times. The subjects were divided into 4 groups using the following age ranges (1) preschool children, age <7 years (mean 5.2±1.5 years; N = 99, 56 females); (2) primary school children, 7 ≤ age <10 years (mean 8.5±0.8 years; N = 111, 59 females); (3) pre-adolescence, 10 ≤ age <14 years (mean 11.8±1.1 years; N = 93, 49 females); (4) adolescence, age ≥ 14 years (mean 17.8±2.2 years, N = 106, 56 females). This age classification was adopted from clinical findings [24] and reflects an age based classification using clinical parameters.

None of the subjects had any history of cardiovascular disease. In 4 subjects there were diseases (e.g. bronchial asthma) which may have influenced cardiovascular parameters. Five subjects were taking medication for attention-deficit hyperactivity disorder and 4 subjects were receiving naturopathic treatment. A post-hoc analysis comparing the results of the entire group and the results of the groups without the above-mentioned subjects showed only negligible differences. Hence, all subjects were considered in this study.

All recordings were obtained during regular school days. This represents a quasi-controlled condition although we did not standardize the daily schedule. The daily routine was structured such that all subjects had to be at school or preschool on time in the morning (between 8 and 9 am). While at school the levels of physical activity varied between low (e.g. sitting during classes) and moderate (e.g. walking during breaks). In the afternoon the activities were less structured. Many subjects had to do homework with corresponding low physical activity. Bedtime in the evening was determined by the need to get up in time the next morning. Taken together, the activities during the day were adjusted to the requirements of dealing with a regular school day. Individual preferences of daily activities (and also sleeping habits) were subordinated to this requirement.

### ECG recordings

24 hour-Holter ECGs were recorded under everyday conditions. In 391 subjects the duration of the ECG recording was ≥16 hours (mean 22.3±1.5 hours). In the other subjects (N = 18) the duration was 4.2 hours (±0.6 hours) because their age was <4 years. In these subjects the ECGs were recorded only while in preschool (including an after-lunch nap of at least 0.5 hour). HR parameters during the nap were then used as a substitute for nighttime values.

The digital Holter device (Medilog MK3, Schiller-Engineering, Graz, Austria) permitted an internal R-peak detection by the device with a precision of <1 ms (sampling rate: 4096 Hz). The ECG (at a sampling rate of 256 Hz) as well as the automatically identified times of the R-peaks were transferred to a PC. The times of the devices' automatically identified R-peaks were inspected manually. In case of ventricular extrasystoles, artefacts and premature beats the timings of the related beats were marked accordingly (<0.5% of all recorded R-peaks). Times of normal beats that were not correctly identified by the device's internal R-peak detection were corrected manually on the basis of the saved ECG. Hence, the timings of these beats had a precision of 4 ms (<1.5% of all detected R-peaks).

### Standard HRV

The mean of normal-to-normal R-R intervals (mNN) as well as its accompanying standard deviation (SDNN) were calculated as measures in the time domain. In the frequency domain, the extent of very low frequency oscillations (VLF: 0.0033 to 0.04 Hz), low frequency oscillations (LF: 0.04 to 0.15 Hz) and high frequency oscillations (HF: 0.15 to 0.4 Hz) were quantified on the basis of the re-sampled R-R interval series (sampling rate: 4 Hz) using the Fast Fourier transformation [4]. In addition, the ratio LF/HF was calculated as an estimator of the balance between oscillations of the sympathetic and parasympathetic branch of the ANS. The frequency domain measures were calculated for each consecutive 1-hour epoch of the recording. Daytime and nighttime values of the time and frequency domain measures were obtained by calculating the average values from 0 am to 6 am (nighttime) and from 9 am to 6 pm (daytime). Furthermore, the extent of ultra low frequency oscillations (ULF: ≤0.0033 Hz) and also VLF oscillations were quantified using the R-R interval series from the entire recording. All spectral measures except LF/HF were expressed in units of ms^2^ because the total power was equivalent to the variance of the R-R interval series. These measures had to be transformed by taking the natural logarithm (indicated by ‘ln ms^2^’) to yield normal distributions. LF/HF was also transformed by taking the natural logarithm to improve the skewed distribution caused by calculation of the ratio.

### Scaling and complexity of HR

**Figure 1:**
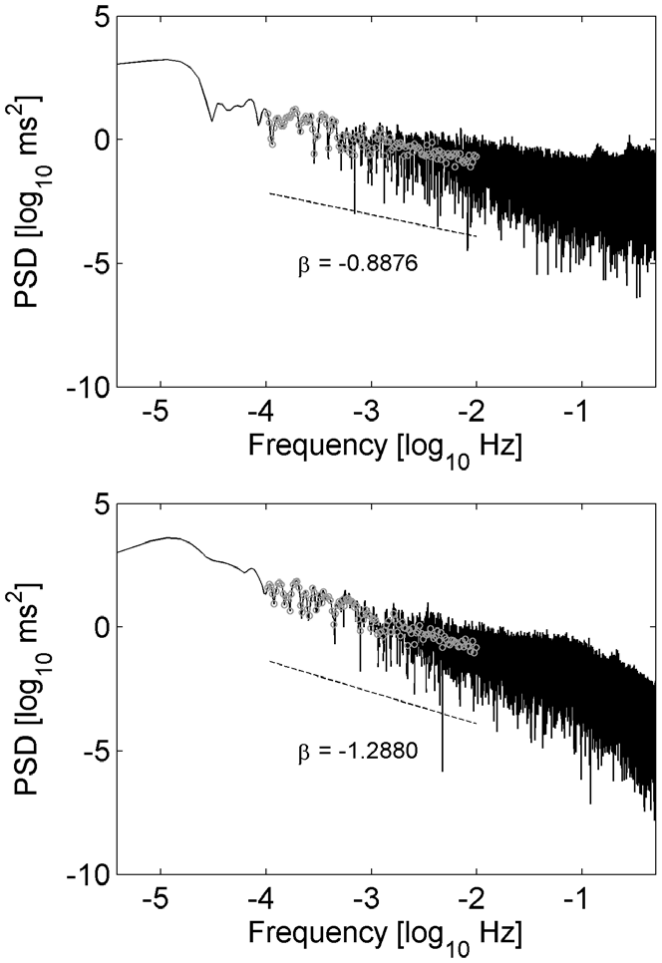
Examples of the power spectral density (PSD) of a 24 h R-R interval series in a double logarithmic plot (decadic logarithm as indicated by ‘log_10_’). The slope β denotes the slope of the linear regression calculated using the averaged PSD (grey circles; frequency range: 10^−4^ to 10^−2^ Hz). Top: male subject, age 7.9 years; bottom: male subject, age 18.1 years.

The R-R interval series exhibits temporal self similarity on different time scales (fractal scaling-like properties) analogous to spatial self similarity on different length scales. [8] Long-term fractal scaling-like correlation properties of the entire R-R interval series were quantified by the slope of the linear, very low frequency part of the power spectrum, log(power) vs. log(frequency), of the R-R interval series. This part indicates a 1/f^β^ relationship between power and frequency. A slope β = 0 corresponds to white noise (no correlations and scaling) whereas Brownian motion (showing correlations and scaling on different scales) leads to β = −2. The slope β was calculated using a smoothed power spectrum in the frequency range 0.0001 to 0.01 Hz (see Figure 1) [25]. Intermediate-term and short-term fractal scaling-like correlation properties of the R-R interval series were quantified using DFA. The fluctuations of the integrated and detrended R-R interval series depend on the chosen window size [7]. If log(fluctuations) is plotted against log(window size) linear relationships appear in two different regions. The slope of the linear relationship is referred to as scaling exponents α_1_ (windows size: 4 to 11 beats) and α_2_ (windows size: >11 beats) indicating short and intermediate-term correlations. These measures were calculated using epochs with 8000 consecutive R-R intervals of the entire recordings. The average values of all epochs were used.

Complexity was quantified using Approximate Entropy (ApEn), a measure of regularity of a time series [5], [26]. Given an R-R interval series of length N, 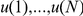, sequences of vectors 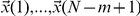 with m components are formed by defining 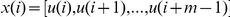. The distance 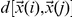 between two vectors 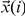 and 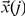 is defined by the maximum difference of all their scalar components as: 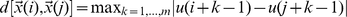. For each vector 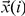 the number of neighbours 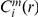 closer than the radius r is calculated: 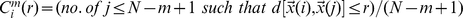. The difference between the average logarithmic 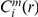 and 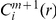 represents ApEn: 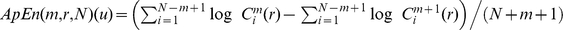. Hence, if an R-R interval series is regular 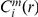 and 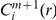 are very similar and ApEn is low whereas irregular sequences are characterized by diverging values of 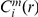 and 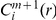, i.e. increased values of ApEn. Hence, the more irregular (complex) the R-R interval series the greater the ApEn values. ApEn was also calculated from consecutive epochs comprising 8000 R-R intervals. The parameters were set to 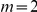 and 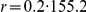 (20% of the average SDNN across all recordings to enable comparability between all recordings). Subsequently, average nighttime (0 am to 6 am) and daytime (9 am to 6 pm) values were calculated.

We also calculated Sample Entropy as a another measure of regularity that remedies some flaws in the ApEn algorithm [6]. The results of Sample Entropy with respect to the changes during childhood and adolescence were very similar to those of ApEn. Hence, we only report on the results of ApEn.

### Statistics

The dependency of each measure on age was modelled by a regression analysis using a 3^rd^ order polynomial. This way a functional dependency with respect to age-related changes could be estimated. The significance of this modelling was calculated using the F-statistic.

The results of the groups are presented as mean ± sd. Comparisons between the four groups were carried out using the 1-way ANOVA procedure. The post-hoc comparisons were Bonferroni corrected. Student's t-test was used to compare results of males and females as well as daytime and nighttime values. Pearson's correlation coefficient *r* was used if a relationship between a variable and age was quantified. A value p<0.05 was considered statistically significant.

## Results

**Figure 2:**
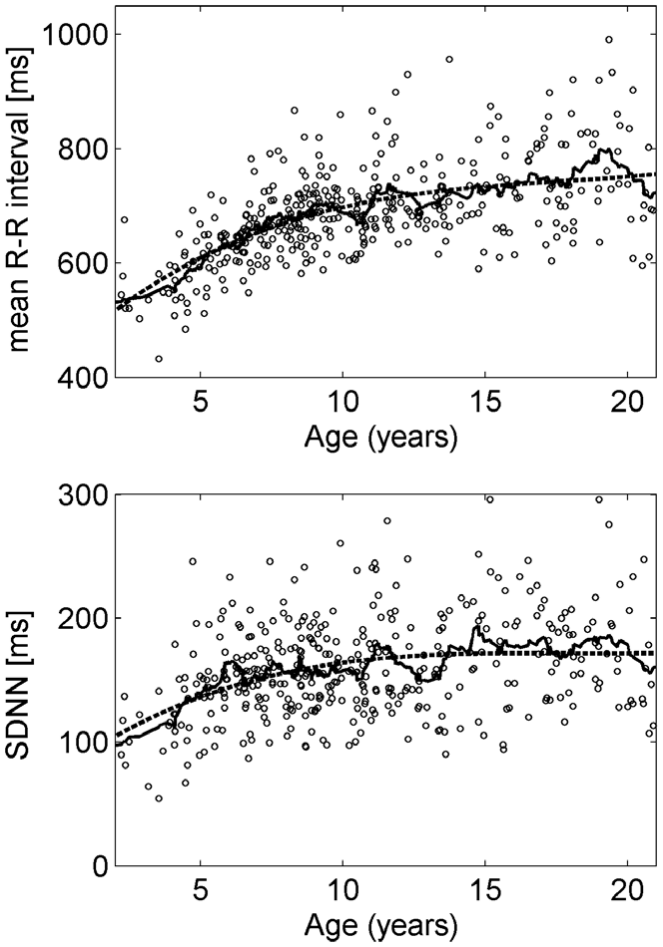
Course of the mean R-R interval and its accompanying standard deviation (SDNN) as basic time domain measures during childhood and adolescence. The circles represent 24 h averages. Solid and dashed lines show moving average and the fitted polynomial model of order 3, respectively.

**Figure 3:**
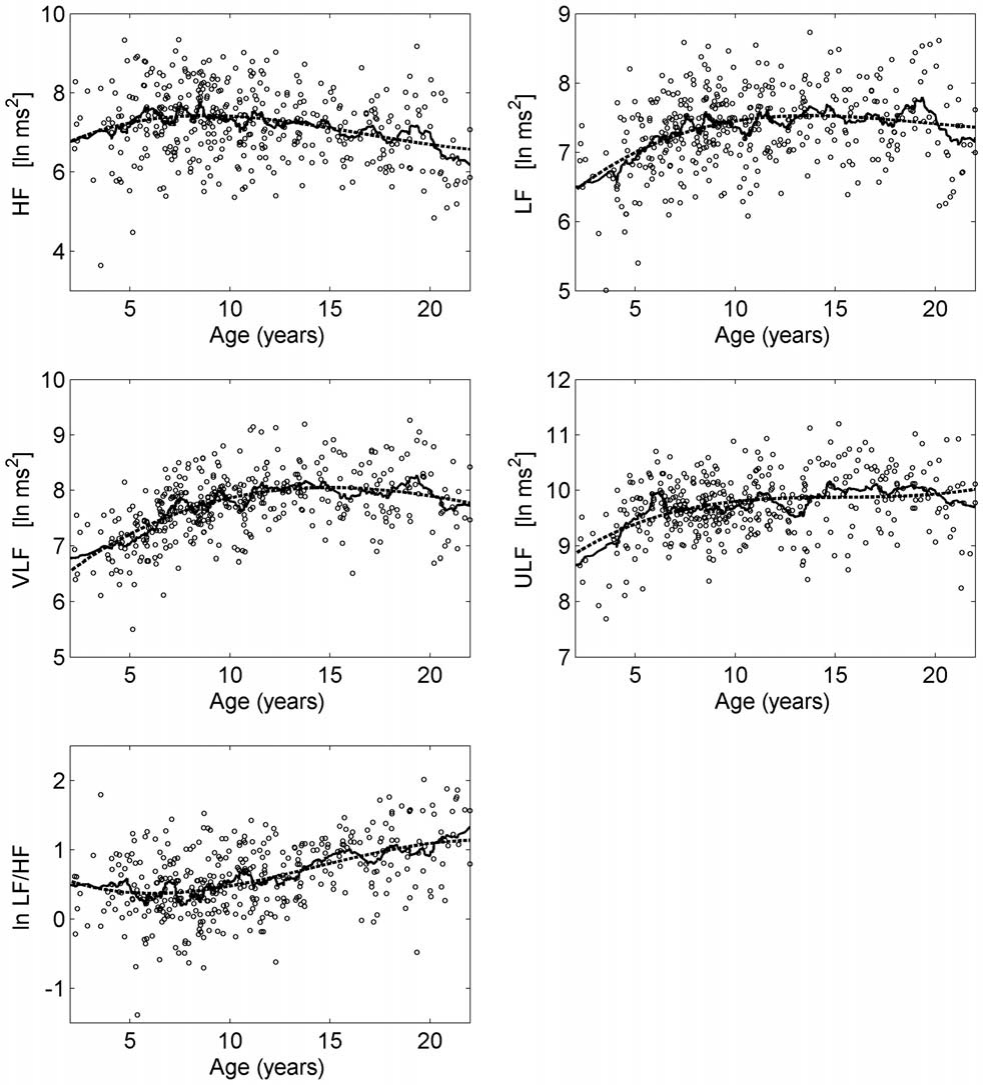
Course of the frequency domain measures during childhood and adolescence. HF – high frequency component, LF – low frequency component, VLF – very low frequency component, ULF – ultra low frequency component, ln ms^2^ – natural logarithm of the absolute values in ms^2^, ln LF/HF - natural logarithm of the ratio LF/HF. The circles represent 24 h averages. Solid and dashed lines show moving average and the fitted polynomial model of order 3, respectively.

All measures reflecting linear and nonlinear dynamics of the R-R interval series showed variations during childhood and adolescence (see Figures 1, 2, 3). In each figure the diagrams also show the moving average (window length: 21 data points, solid line) and the model (dashed line). In all cases the model resembled the moving average indicating that the model was sufficient to explain the average development during childhood and adolescence. The largest adjusted coefficients of determination were obtained for mean R-R interval (R^2^ = 0.40), VLF (R^2^ = 0.33), DFA α_1_ (R^2^ = 0.29) and the slope β (R^2^ = 0.23). ApEn (R^2^ = 0.05) and HF (R^2^ = 0.07) had the lowest adjusted coefficient of determination. Nevertheless, the model was significant for all measures.

### Time and frequency domain measures

**Figure 4:**
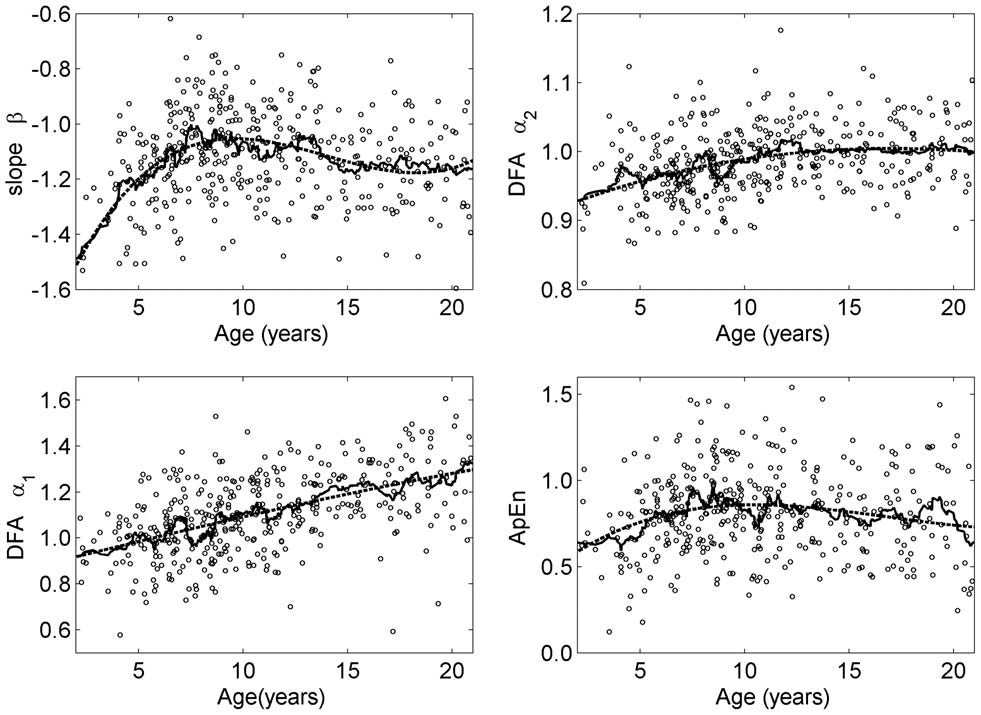
Course of the fractal and complexity measures during childhood and adolescence. Slope β – long-term fractal scaling properties, DFA α_2_ – intermediate-term fractal scaling properties, DFA α_1_ – short-term fractal scaling properties, ApEn - complexity. The dots represent 24 hour averages. Solid and dashed lines show moving average and the fitted polynomial model of order 3, respectively.

The results of the four age groups were as follows. The mean R-R interval (mNN) was lowest in the group <7 years, as was SDNN (mNN: 607 ms, SDNN: 139 ms; see Figure 4). The longest mNN and the greatest SDNN were observed in the group ≥14 years (mNN: 745 ms, SDNN: 175 ms). The subjects <10 years showed the strongest relationship between age and mean R-R interval (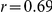). SDNN and age correlated strongest in the group <7 years (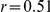).

In the frequency domain the ULF component was lowest for the group <7 years (9.46 ln ms^2^) and largest for the group ≥14 years (9.98 ln ms^2^). The strongest correlation between ULF and age was found in the group <7 years (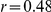). The VLF and LF components were also lowest in the group <7 years (VLF: 7.18 ln ms^2^, LF: 6.98 ln ms^2^). However, the increase of the VLF component was slower compared to ULF and continued up to the age of 10 (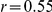). The increase of LF also continued up to the age of 10 but the strongest relationship with respect to age was observed in the group <7 years (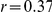). Interestingly, the HF component was constant up to the age of 13 years (7.24 ln ms^2^). In the group ≥14 years HF decreased (6.90 ln ms^2^). There was no correlation to age. LF/HF was constantly low up to the age of 10 (0.41). It increased with progressing age and was largest for the group ≥14 years (0.98). Hence, the strongest correlation with age was observed for subjects ≥7 years (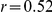). As expected, the course of LF/HF with progressing age reflects a combination of the course of LF and the course of HF.

### Scaling and complexity of the R-R interval series

**Figure 5:**
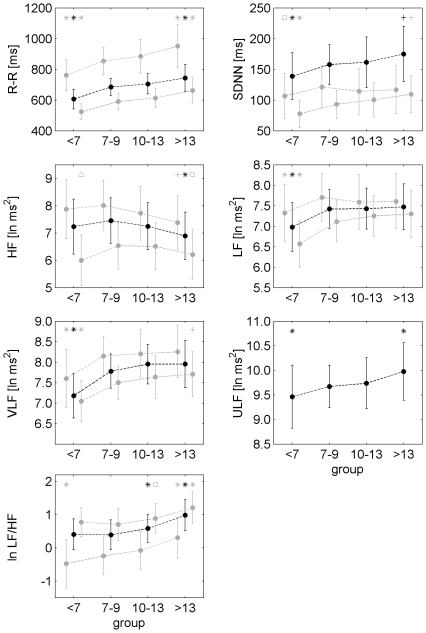
Comparison of different age groups with respect to time and frequency domain measures. R-R – mean R-R interval, SDNN – standard deviation of R-R interval series, HF – high frequency component, LF – low frequency component, VLF – very low frequency component, ULF – ultra low frequency component, ln ms^2^ – natural logarithm of the absolute values in ms^2^, ln LF/HF - natural logarithm of the ratio LF/HF. Each group shows three values: 24 h averages are plotted in black, night-time (left of black dot) and wake time averages (right of black dot) are plotted in grey. Note that ULF can only be calculated for the entire recording. The frequency domain measures were transformed by taking the natural logarithm to yield normal distributions (indicated by ‘ln ms^2^’). The symbol above a value/dot refers to comparisons within the same time period (24 h, night-time or wake time).* Group differed from 3 other groups. + Group differed from group <7 years and group 7 to 9 years. □ Group differed from group 7 to 9 years. ▵ Group differed from group 7 to 9 years and group 10 to 13 years.

The fractal scaling measure DFA α_1_ was lowest for the group <7 years (0.99, see Figure 5) and highest in the group ≥14 years (1.24). DFA α_2_ was lower for the subjects <10 years (0.96) compared to the subjects ≥10 years (1.00). Both, α_1_ and α_2_, showed only a weak relationship to age for the subjects <7 years (α_1_: 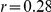; α_2_: 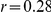). The slope β showed unexpected variations with age. The slope was lowest in the groups <7 years and ≥14 years (−1.21) and increased in the subjects 7 to 12 years (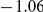). The relationship between β and age was 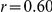 for the group <7 years. For the subjects ≥7 years the relationship was 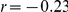.

ApEn also showed unexpected variations with age. ApEn was lowest for the groups <7 years and ≥14 years (0.74) whereas complexity increased in the group 7 to 9 years (0.88). Hence, the relationship between ApEn and age was 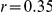 for the subjects <10 years (group <7 years and group 7 to 9 years). Furthermore, a negative correlation was observed for the subjects ≥7 years (group 7 to 9 years, group 10 to 13 years and group ≥14 years; 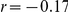).

### Day-night differences

The standard measures of the R-R interval dynamics showed a difference between daytime and nighttime values (cf. Figure 5, all differences p<0.001 except for SDNN in the group ≥14 years: p<0.05). mNN was longer during nighttime compared to daytime in all age groups. Accordingly, SDNN was higher during nighttime in all groups. In the frequency domain all measures showed day-night differences. Generally, nighttime values were higher than daytime values. Nighttime and daytime values of the mNN, VLF and LF showed similar variations with age compared to the 24 h averages of these measures. Nighttime SDNN only differed between the group <7 years and the group 7 to 9 years. Nighttime HF decreased with age whereas daytime HF was lower for the groups <7 years and ≥14 years compared to the group 7 to 9 years. Hence, the decrease of the 24 h average HF in the group ≥14 years seems to be determined largely by the decrease of nighttime HF. As expected LF/HF was always lower during nighttime compared to daytime.

**Figure 6:**
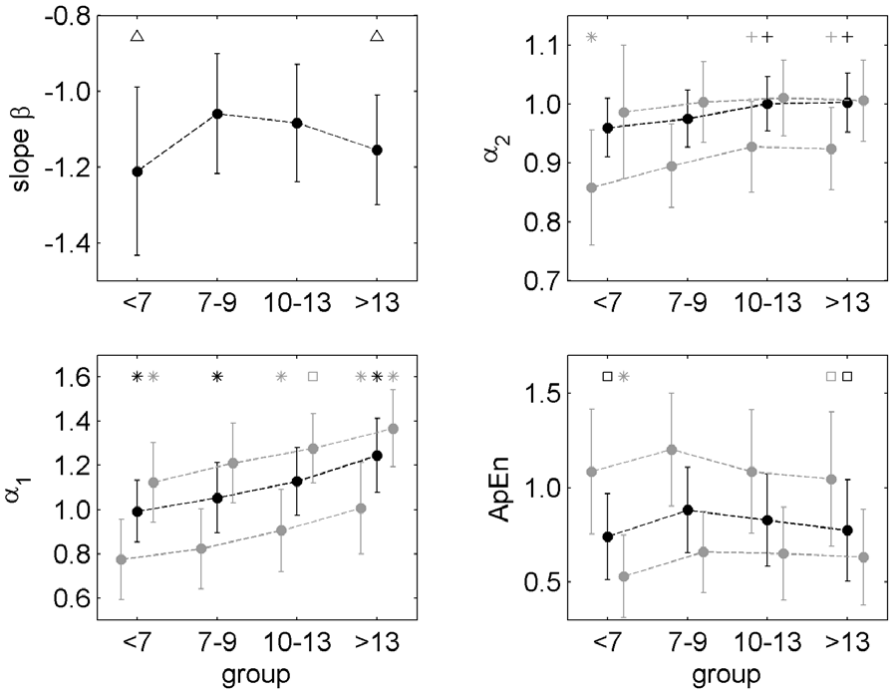
Comparison of different age groups with respect to scaling and complexity measures. Slope β – long-term fractal scaling properties, DFA α_2_ – intermediate-term fractal scaling properties, DFA α_1_ – short-term fractal scaling properties, ApEn - complexity. Each group shows three values: 24 h averages are plotted in black, night-time (left of black dot) and wake time averages (right of black dot) are plotted in grey. Note that the slope β can only be calculated for the entire recording. The symbol above a value/dot refers to comparisons within the same time period (24 h, night-time or wake time). * Group differed from 3 other groups. + Group differed from group <7 years and group 7 to 9 years. □ Group differed from group 7 to 9 years. ▵ Group differed from group 7 to 9 years and group 10 to 13 years.

All measures reflecting fractal properties and complexity differed during daytime and nighttime in all groups (cf. Figure 6, p<0.001). DFA α_1_ and α_2_ were lower during nighttime. During nighttime α_1_ was lower in the group <7 years compared to the group ≥14 years whereas daytime values increased during the same time span. DFA α_2_ during nighttime also increased over the entire time span whereas α_2_ during daytime was constant. ApEn was lower during daytime. Irregularity was higher in the group 7 to 9 years compared to the group ≥14 years. During daytime the irregularity was lowest for the group <7 years and increased with progressing age.

### Gender differences

In case of differences between male and female subjects in the time and frequency domain male subjects always had higher values. In the group 7 to 9 years males had higher values in the following measures: mNN (705 vs. 670 ms, p<0.001), SDNN (167 vs. 149 ms, p<0.001), LF (7.54 vs. 7.32 ln ms^2^, p<0.05), VLF (7.90 vs. 7.68 ln ms^2^, p<0.01) and ULF (9.81 vs. 9.56 ln ms^2^, p<0.01). The males of the group ≥14 years had higher values with respect to mNN (775 vs. 717 ms, p<0.001), SDNN (193 vs. 160 ms, p<0.001), HF (7.14 vs. 6.90 ln ms^2^, p<0.001), LF (7.76 vs. 7.22 ln ms^2^, p<0.001), VLF (8.22 vs. 7.71 ln ms^2^, p<0.001) and the ULF (10.15 vs 9.82 ln ms^2^, p<0.01). LF/HF did not show any difference between male and female subjects although LF showed gender differences in the age groups 7 to 9 years and ≥14 years and also HF showed gender differences for the age group ≥14 years.

With respect to fractal and complexity measures the male subjects in the group 10 to 13 years showed higher values of the slope β (−1.04 vs. −1.12, p<0.05). The males of the group ≥14 years also had a higher slope β (−1.09 vs. −1.21, p<0.001) and a larger ApEn (0.86 vs. 0.69, p<0.01).

## Discussion

The main finding is that complexity and scaling properties show unexpected variations during childhood and adolescence. These measures do not show a monotonic increase as might be expected from e.g. the monotonic increase of the average R-R interval. With respect to the fractal scaling properties it should be noted that a slope β = 0 and DFA α = 0.5 (for both, α_1_ and α_2_) indicate a time series without correlations (white noise) whereas correlations in the time series lead to β < 0 and α>0.5 (cf. reference [7]). Furthermore, α = 1 and β = −1 indicate that the power spectrum of the R-R interval series shows a 1/f relationship. This property is an indicator of scale-free dynamics, i.e. the similarity of time series properties on different time scales, as e.g. observed in healthy states [8]. Interestingly, long-term fractal scaling properties (slope β) in preschool children (age <7 years) and adolescents (age >13 years) deviate from 1/f behaviour and tend toward 1/f^2^ behaviour indicating stronger correlations. On the other hand, during primary school age (age 7 to 9 years) and pre-adolescence (age 10 to 13 years) this property is close to 1/f behaviour. These results are also supported by the regression analysis. The finding in the age range 7 to 13 years is unexpected because the age-related course in adults tends in the opposite direction (towards 1/f^2^ behaviour) with increasing age [10]. This tendency is even more pronounced in patients after myocardial infarction [27].

The 1/f behaviour of intermediate-term fractal scaling properties α_2_ was not substantially altered whereas α_1_ indicated a change from 1/f behaviour toward 1/f^2^ behaviour of short-term fractal scaling properties from preschool children to adolescence.

ApEn was lower in preschool children and during adolescence compared to primary school children. Hence, the R-R interval time series was more regular (more predictable) in the former. During primary school age ApEn increased and the R-R interval time series was more irregular (less predictable). We note that these differences were observed although the qualitative changes as expressed by the regression analysis were weak. Taken together, ApEn and the slope β show a similar variation with age although these measures reflect properties on different time scales: ApEn essentially reflects complexity on a short time scale (a few heartbeats) whereas β quantifies long-term fractal properties. Given that the lower the slope β the more regular the R-R interval dynamics [7], this similarity may reflect a relationship between these two measures.

The average R-R interval increased during childhood up to the age of 9 years as expected. It was constant during primary school age and puberty. These age related variations as well as the large variations in the respective age groups are in agreement with the results of a large sample under controlled conditions reported previously [28]. During adolescence the average R-R interval increased again. This course was also apparent in SDNN in the time domain and ULF in the frequency domain whereas LF and VLF increased only up to the age of 9 years. The findings with respect to the mean R-R interval, SDNN, VLF and LF are in accordance with other studies [15], [16], [29], [30]. The HF component was constant up to puberty and decreased during adolescence. The age related changes during puberty and adolescence have already been reported [29], [31] whereas an increase of the HF component has been reported for primary school children by one study [29]. In a previous study the ratio LF/HF decreased during the first two years after birth [15]. Subsequently, LF/HF increased with progressing age similarly to the results in the present study. We were not able to reproduce the results of the very young subjects because in this age group the number of subjects was too low.

Day-night differences showed that the average R-R interval was longer during nighttime sleep. At the same time, the time and frequency domain measures reflect a greater cardiac variability. The complexity of the R-R interval series also increased at night indicating a greater irregularity. This was to be expected because complexity increases if the average R-R interval increases [2], [3], [8]. As short- and intermediate-term fractal scaling properties during nighttime were lower, the R-R interval series showed less correlation. The age-related variations of daytime and nighttime values were similar to those calculated from the entire 24 h recording for most measures. The most interesting deviation was observed for the HF component. During daytime the HF component was lowest in preschool children and during adolescence whereas it increased in the intermediate period, similar to findings in another study [29].

Most gender differences were observed for primary school children and for adolescents. In these two groups the following differences were observed. The time and frequency domain measures (average R-R interval, SDNN, ULF, VLF and LF; HF only for adolescents) were lower for female subjects. Complexity was also lower in female subjects during adolescence showing that the R-R interval dynamics was more regular in these subjects. This result is in accordance with findings showing that reduced R-R variations in patients with heart failure are accompanied by a reduction of complexity and chaotic features [2], [3], [8]. Long-term fractal scaling properties were different during pre-adolescence and adolescence. Female subjects showed an increased slope β, i.e. the R-R interval dynamics were closer to 1/f behaviour. Taken together, gender differences are obviously age related. They reflect gender specific aspects of maturation.

Keeping in mind that the linear measures should be cautiously interpreted in terms of activity of both branches of the ANS [1], [4] these results suggest that in preschool children in particular the oscillations of sympathetic activity increase (as indicated by the increase of VLF and LF) whereas the oscillations of parasympathetic activity are constant (as indicated by HF). The ratio LF/HF as a quantity reflecting this balance also shows an increase with progressing age indicating a shift towards a predominance of sympathetic modulations. Regardless of the oscillations of the parasympathetic branch, the average tone of this branch increases which is reflected by the increase of the average R-R interval with progressing age. However, it remains to be clarified how these processes are linked to the age-related variations of long-term fractal scaling properties and complexity.

The age-related variations of the different linear and nonlinear properties of R-R interval dynamics are obviously related to different stages of somatic maturation during childhood and adolescence. Both age-related changes as well as gender differences of R-R interval dynamics are likely linked to (pre-) pubertal hormonal changes [17] because hormones such as estrogen and progesterone as well as substantial changes of hormonal levels during induction of ovulation, onset of menopause or estrogen replacement therapy have been associated with alterations of R-R interval dynamics [32]–[34]. The present results with respect to long-term fractal scaling properties and complexity suggest that pre-puberty and adrenarche (age 7 to 9 years) also have a specific impact on R-R interval dynamics. Adrenarche and pre-puberty are characterized by an increased production of androgens, particularly dehydroepiandrosterone from the adrenal zona reticularis [35]. These processes of maturation are accompanied by a vulnerability with respect to the onset of different diseases [19]–[23]. Hence, the results of this study may point to a complex physiological mechanism of the ANS linked to the gordian development and maturation from neonate to old age. A better understanding of the functioning of the ANS and its interaction e.g. with the central nervous system, hormone production and extrinsic factors [36] remains a challenging and important question of clinical significance.

### Limitations

24 h ECG recordings under normal everyday conditions were deemed to be the appropriate choice for reliable calculation of the different measures for quantifying R-R interval dynamics over various time scales and behavioural states. The physiological interpretation of the different measures calculated from such recordings is naturally limited. The enhancement of physiological interpretability only would have been possible by defining conditions that affect the R-R interval dynamics (e.g. respiratory rate, physical activity) [37]. However, such restrictions would have been inappropriate for long-term recordings, particularly for children and adolescents. Nevertheless, nighttime and daytime averages still contain a large amount of information that can be used to specify characteristics of ‘normal conditions’.

The activities of the subjects during the recording of the ECG were determined by the need to cope with a regular school day, representing a quasi-controlled condition. Hence, the level of daytime activities varied from relatively lower levels of physical activity (and higher levels of mental activity) in the morning to higher levels of physical activity in the afternoon. These variations affect different parameters and, hence, may have contributed to the large variance of the different 24 h and daytime parameters (cf. Figures 2, 3, 4). However, it is not clear whether this caused an increase of e.g. LF or VLF or a decrease of parameters. It should be noted that in a previous study using controlled conditions the variance of the average heart rate of each age group was also large [28]. A review on heart rate during childhood and adolescence found similar results [38]. Hence, the large variance may reflect differences of the individual characteristics of cardiovascular control rather than differences of activity.

Children <4 years (16 out of 99 subjects in the group <7 years, cf. Methods section) were monitored only while in preschool, including an after-lunch nap, due to restrictions imposed by the ethics committee. Preschool activities (and resting periods) are arranged according to the needs of the children at this age. They may be different from activities in the family environment. However, the average R-R interval and the variance in this group is in agreement with the corresponding age group of a review [38] and a previous study [28].

### Conclusion

Maturation of the ANS during childhood and adolescence as represented by cardiac activity is not a trivial function of age. Measures reflecting complexity and long-term fractal scaling properties reveal unexpected changes during childhood and adolescence. These changes are different from those found in the linear measures. Hence, the effect of age on R-R interval dynamics has to be taken into account for the entire lifespan [10] as well as during childhood and adolescence.

## References

[1] Malik M, Camm AJ (1995). Heart rate variability..

[2] Poon CS, Merrill CK (1997). Decrease of cardiac chaos in congestive heart failure.. Nature.

[3] Ivanov PC, Amaral LAN, Goldberger AL, Havlin S, Rosenblum MG (1999). Multifractality in human heartbeat dynamics.. Nature.

[4] Task Force of the European Society of Cardiology and the North American Society of Pacing and Electrophysiology (1996). Heart rate variability: standards of measurement, physiological interpretation, and clinical use.. Circulation.

[5] Pincus SM, Goldberger AL (1994). Physiological time-series analysis: What does regularity quantify?. Am J Physiol.

[6] Richman JS, Moorman JR (2000). Physiological time-series analysis using approximate entropy and sample entropy.. Am J Physiol Heart Circ Physiol.

[7] Peng CK, Havlin S, Stanley H, Goldberger AL (1995). Quantification of scaling exponents and crossover phenomena in nonstationary heartbeat time series.. Chaos.

[8] Goldberger AL, Amaral LA, Hausdorff JM, Ivanov PC, Peng CK (2002). Fractal dynamics in physiology: alterations with disease and aging.. Proc Natl Acad Sci U S A.

[9] Bassingwaighte JB, Liebovitch LS, West BJ (1994). Fractal physiology..

[10] Pikkujamsa SM, Makikallio TH, Sourander LB, Raiha IJ, Puukka P (1999). Cardiac interbeat interval dynamics from childhood to senescence - Comparison of conventional and new measures based on fractals and chaos theory.. Circulation.

[11] Umetani K, Singer DH, McCraty R, Atkinson M (1998). Twenty-four hour time domain heart rate variability and heart rate: Relations to age and gender over nine decades.. J Am Coll Cardiol.

[12] Beckers F, Verheyden B, Aubert AE (2006). Ageing and non-linear heart rate control in a healthy population.. Am J Physiol Heart Circ Physiol.

[13] Iyengar N, Peng CK, Morin R, Goldberger AL, Lipsitz LA (1996). Age-related alterations in the fractal scaling of cardiac interbeat interval dynamics.. Am J Physiol.

[14] Massin MM, Withofs N, Maeyns K, Ravet F, Gerard P (2001). Normal ranges for the variability in heart rate in young infants while sleeping.. Cardiol Young.

[15] Massin M, von Bernuth G (1997). Normal ranges of heart rate variability during infancy and childhood.. Pediatr Cardiol.

[16] Silvetti MS, Drago F, Ragonese P (2001). Heart rate variability in healthy children and adolescents is partially related to age and gender.. Int J Cardiol.

[17] DiVall SA, Radovick S (2008). Pubertal development and menarche.. Ann N Y Acad Sci.

[18] Shirtcliff EA, Dahl RE, Pollak SD (2009). Pubertal development: correspondence between hormonal and physical development.. Child Dev.

[19] Masten AS (2004). Regulatory processes, risk, and resilience in adolescent development.. Ann N Y Acad Sci.

[20] Patton GC, Viner R (2007). Pubertal transitions in health.. Lancet.

[21] Kessler RC, Amminger GP, Aguilar-Gaxiola S, Alonso J, Lee S (2007). Age of onset of mental disorders: a review of recent literature.. Curr Opin Psychiatry.

[22] Shaw J (2007). Epidemiology of childhood type 2 diabetes and obesity.. Pediatr Diabetes.

[23] Subbarao P, Mandhane PJ, Sears MR (2009). Asthma: epidemiology, etiology and risk factors.. CMAJ.

[24] Kliegman RM, Behrman RE, Jenson HB, Stanton BF (2007). Nelson textbook of pediatrics..

[25] Bigger JT, Steinman RC, Rolnitzky LM, Fleiss JL, Albrecht P (1996). Power law behavior of RR-interval variability in healthy middle-aged persons, patients with recent acute myocardial infarction, and patients with heart transplants.. Circulation.

[26] Pincus S (1995). Approximate entropy (ApEn) as a complexity measure.. Chaos.

[27] Makikallio TH, Hoiber S, Kober L, Torp-Pedersen C, Peng CK (1999). Fractal analysis of heart rate dynamics as a predictor of mortality in patients with depressed left ventricular function after acute myocardial infarction.. Am J Cardiol.

[28] Davignon A, Rautaharju P, Boisselle E, Soumis F, Megelas M (1980). Normal ECG standards for infants and children.. Ped Cardiol.

[29] Goto M, Nagashima M, Baba R, Nagano Y, Yokota M (1997). Analysis of heart rate variability demonstrates effects of development on vagal modulation of heart rate in healthy children.. J Pediatr.

[30] Kazuma N, Otsuka K, Wakamatsu K, Shirase E, Matsuoka I (2002). Heart rate variability in normotensive healthy children with aging.. Clin Exp Hypertens.

[31] Finley JP, Nugent ST (1995). Heart rate variability in infants, children and young adults.. J Auton Nerv Syst.

[32] Christ M, Seyffart K, Wehling M (1999). Attenuation of heart-rate variability in postmenopausal women on progestin-containing hormone replacement therapy.. Lancet.

[33] Bai X, Li J, Zhou L, Li X (2009). Influence of the menstrual cycle on nonlinear properties of heart rate variability in young women.. Am J Physiol Heart Circ Physiol.

[34] Weissman A, Lowenstein L, Tal J, Ohel G, Calderon I (2009). Modulation of heart rate variability by estrogen in young women undergoing induction of ovulation.. Eur J Appl Physiol.

[35] Auchus RJ, Rainey WE (2004). Adrenarche - physiology, biochemistry and human disease.. Clin Endocrinol (Oxf).

[36] Thayer JF, Hansen AL, Saus-Rose E, Johnsen BH (2009). Heart rate variability, prefrontal neural function, and cognitive performance: The neurovisceral integration perspective on self-regulation, adaptation, and health.. Ann Behav Med.

[37] Grossman P, Wilhelm FH, Spoerle M (2004). Respiratory sinus arrhythmia, cardiac vagal control and daily activity.. Am J Physiol Heart Circ Physiol.

[38] Fleming S, Thompson M, Stevens R, Heneghan C, Pluddemann A (2011). Normal ranges of heart rate and respiratory rate in children from birth to 18 years of age: a systematic review of observational studies.. Lancet.

